# Integrated OMICS tools for personalised medicine

**DOI:** 10.1186/1129-2377-16-S1-A9

**Published:** 2015-09-28

**Authors:** Marina Borro, Giovanna Gentile, Luana Lionetto, Maurizio Simmaco

**Affiliations:** Advanced Molecular Diagnostics Unit, Sant'Andrea Hospital, Sapienza University of Roma, Rome, Italy

System-wide adoption of Personalised Healthcare requires an active and flexible but highly integrated infrastructure, joining many different competences and technologies and allowing continuous upgrading, also through self-learning processes. In this system, clinics and diagnostics would no longer be partitioned, according to a stepwise scheme, from other disciplines as basic science, information technologies, ethics and policies. Each player constitutes a node of a cabled network, where input and output from each node are automatically transferred to all nodes, to systematically retune and coordinate the global activity. Since 2005 the Sant'Andrea Hospital of Rome is an in-house built model of Personalised Healthcare Service, which auto-catalytically drives its own development, which may furnish good evidence that translation of Personalised Medicine into clinical practice is not so elusive. The interaction between usually distant academic departments and wards (as Biochemistry, Internal Medicine, Psychiatry, Oncology), favoured by a farsighted management of hospital resources by the administrators, allowed to create a shared, innovative laboratory, the Advanced Molecular Diagnostics Unit (DiMA). The availability of advanced technologies, as mass spectrometry and medium-to-high throughput DNA analysis paved the way to a “real-time” evaluation of the benefits brought into the “real-world” clinical practice by implementation of new diagnostics aimed to therapy tailoring. This allowed the start up of a health service based on the principles of personalised medicine, in order to optimize the amount of tests necessary to evaluate the patient, to interpret the results correctly and, finally, to plan a personalised therapy and to periodically evaluate and/or modify it, to obtain the best clinical results with the least side effects.

Our *OMICS* platform for personalised medicine offers the following combined approaches:

i) Epigenetics, to obtain information on the regulation of gene expression and to evaluate the methylation profile change during hypomethylation therapies;

ii) Functional genomics, to measure genetic expression in normal and pathologic conditions, in order to define genetic expression profiles;

iii) Structural genomics, which defines genomic differences with clinical impact in the patient populations;

iv) Metabolomics and terapeutic drug managment, to define all molecules of interest in a specific clinical context and the actual drug and metabolite concentration during therapies.

The MIFAR (Metabolismo Integrato FARmaci – drug metabolism integration) including about 60 gene variants has been developed. The data interpretation is ruled out using the Charité Bioinformatic platform (Berlin DE) and allows adaptation of drug therapies to the individual MIFAR profile, improving efficacy and safety of treatments. The DiMA Unit provides pharmacogenomics and theranostics report for at least 5,000 patients/year.
